# Contrast Induced Acute Kidney Injury and its Impact on Mid-Term Kidney Function, Cardiovascular Events and Mortality

**DOI:** 10.1038/s41598-019-53040-5

**Published:** 2019-11-15

**Authors:** Werner Ribitsch, Joerg H. Horina, Franz Quehenberger, Alexander R. Rosenkranz, Gernot Schilcher

**Affiliations:** 1 Clinical Division of Nephrology, Department of Internal Medicine, Medical University of Graz (MUG), Graz, Austria; 2 Institute for Medical Informatics, Statistics and Documentation, Medical University of Graz, Graz, Austria; 3 Intensive Care Unit, Department of Internal Medicine, MUG, Graz, Austria

**Keywords:** Medical research, Acute kidney injury

## Abstract

The existence and clinical relevance of contrast induced acute kidney injury (CI-AKI) is still heavily debated and angiographic procedures are often withheld in fear of CI-AKI, especially in CKD-patients. We investigated the incidence of CI-AKI in cardiovascular high risk patients undergoing intra-arterial angiography and its impact on mid-term kidney function, cardiovascular events and mortality. We conducted a prospective observational trial on patients undergoing planned intra-arterial angiographic procedures. All subjects received standardized intravenous hydration prior to contrast application. CI-AKI was defined according to a ≥25% increase of creatinine from baseline to either 24hrs or 48hrs after angiography. Plasma creatinine and eGFR were recorded from the institutional medical record system one and three months after hospital discharge. Patients were followed up for two years to investigate the long term effects of CI-AKI on cardiovascular events and mortality. We studied 706 (317 female) patients with a mean eGFR of 52.0 ± 15 ml·min^−1^·1.73 m^−2^. The incidence of CI-AKI was 10.2% (72 patients). In 94 (13.3%) patients serum creatinine decreased ≥25% either 24 or 48 hours after angiography. Patients with CI-AKI had a lower creatinine and a higher eGFR at baseline, but no other independent predictors of CI-AKI could be identified. Kidney function was not different between both groups one and three months after discharge. After a two year follow up the overall incidence of cardiovascular events was 56.5% in the CI-AKI group and 58.8% in the Non CI-AKI group (p = 0.8), the incidence of myocardial infarctions, however, was higher in CI-AKI-patients. Overall survival was also not different between patients with CI-AKI (88.6%) and without CI-AKI (84.7%, p = 0.48). The occurrence of CI-AKI did not have any negative impact on mid-term kidney function, the incidence of cardiovascular events and mortality. Considerable fluctuations of serum creatinine interfere with the presumed diagnosis of CI-AKI. Necessary angiographic procedures should not be withheld in fear of CI-AKI.

## Introduction

Contrast enhanced intravascular procedures play a critical role in the diagnosis and treatment of potentially life threatening conditions like coronary heart disease. However, administration of contrast material during angiography can result in contrast induced acute kidney injury (CI-AKI), a serious complication that has been associated with increased intra-hospital morbidity and mortality, persistent decline of kidney function and long-term cardiovascular events^1–3^. Patients with pre-existing chronic kidney disease (CKD) and diabetes are deemed to be especially susceptible for developing CI-AKI^4–6^. Since these patients represent a population at particular high cardiovascular risk, those who would most benefit from angiographic procedures are often excluded from it for fear of CI-AKI. This position is now questioned by recent literature. Several trials have shown that clinically relevant CI-AKI is less frequent than previously assumed^7–9^. However, the picture of CI-AKI is still not clear enough and a lively debate about the existence and the relevance of CI-AKI is still going on^10,11^. Even recently updated guidelines still warn of the hazard of CI-AKI in patients receiving intra-arterial contrast media with an estimated glomerular filtration rate less than 45 ml/min/1.73 m^2^ ^12^. As precise data on the long term effects of CI-AKI are still scarce it was the aim of the present study not only to evaluate the incidence of CI-AKI in cardiovascular high risk patients undergoing intra-arterial angiography but to investigate its impact on mid-term kidney function, cardiovascular events and mortality.

## Materials and Methods

### Study population and study protocol

This single-center, prospective observational cohort study assessed consecutive patients who either underwent an elective percutaneous coronary intervention (PCI) or a percutaneous transluminal angiography/angioplasty of peripheral arteries, renal arteries or carotids. Patients with an eGFR <70 ml/min at study entry originated from a prospective randomized trial previously published in a companion paper^9^. Additionally, we also included patients who had initially been recruited for that randomized trial based on external laboratory results but then had to be excluded due to an eGFR above the allowed threshold of 70 ml/min in the laboratory control. All patients were age >18 years and gave their written informed consent prior to enrollment in the study. We did not include patients on dialysis, patients with evidence of acute kidney injury according to the Acute Kidney Injury Network (AKIN) criteria^13^, pregnancy or administration of iodinated contrast media within 7 days prior to intervention. Patients with emergency procedures were also excluded. The study was conducted in accordance with the Declaration of Helsinki at the Medical University of Graz and was approved by the Institutional Review Board of the Medical University of Graz (IRB00002556, Study Registration Number: 21-278 ex 09/10).

All patients received standardized 0.9% saline 3–5 ml/kgBW per hour for 3 hours prior to angiography for CI-AKI prophylaxis. Monomeric, non-ionic, low osmolar iomeprol (Iomeron 300®, Bracco Vienna, Austria) was used as contrast medium for all study subjects in doses adjusted for body weight. Laboratory tests were assessed at baseline (=day before angiography) and 24–48 hrs after angiography, urine samples were taken at baseline. The primary endpoint was CI-AKI defined according to a ≥25% increase of creatinine from baseline to either 24hrs or 48hrs after angiography as this is a definition very commonly used in CI-AKI studies. If available, plasma creatinine and eGFR were recorded from the institutional medical record system one (m1) and three months (m3) after hospital discharge. Plasma creatinine was measured by the standard Jaffe colorimetric method, Cystatin C by a nephelometric immunoassay and the estimated glomerular filtration rate (eGFR) was calculated via the abbreviated Modification of Diet in Renal Disease (MDRD) study equation^14^. Patients were followed up for two years to investigate the long term effects of CI-AKI on cardiovascular events and mortality. Information about their health status was obtained by direct patient interrogation, from electronic medical records or from the referring primary care physician.

### Statistical analysis

The study was analyzed by nonparametric statistics. Continuous variables were reported as median (minimum - maximum). Wilcoxon’s ranks sum test und Pearson’s chi square test was used for group comparisons of continuous and categorical variables, respectively. The Wilcoxon signed rank and Wilcoxon rank sum tests were applied for the analysis of changes within patients and differences between patients, respectively. The log-rank test was used in survival analysis. P-values below 0.05 were considered statistically significant.

## Results

### Patients

**Figure 1:**
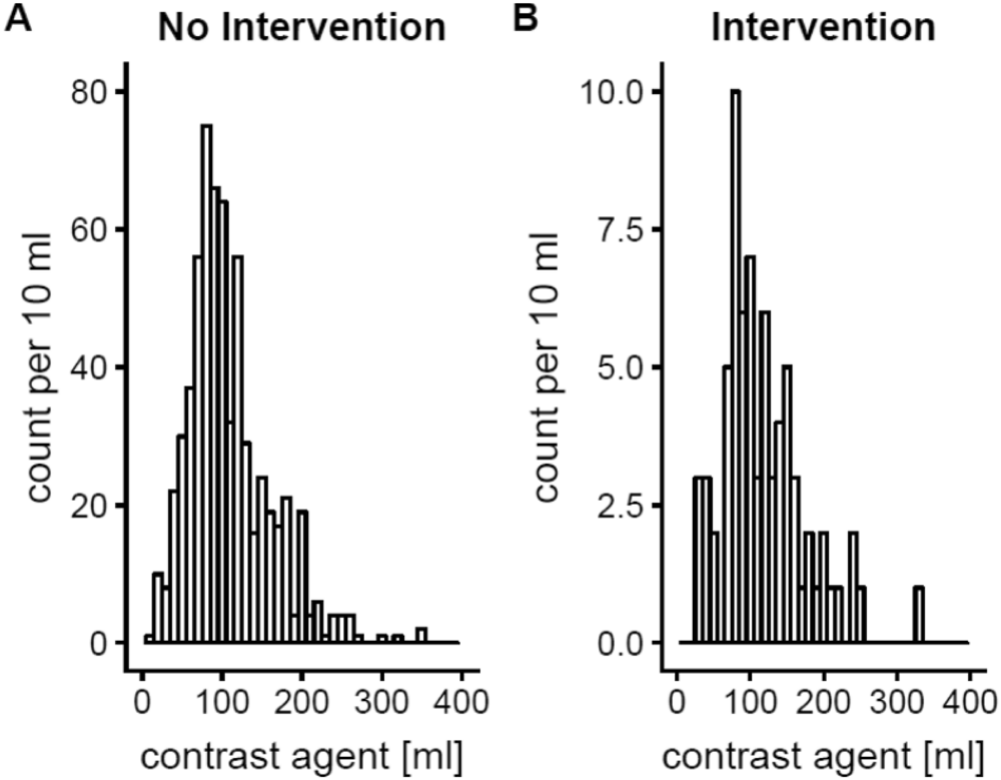
Volumes of contrast media used for angiography (**A**) 95 ml (15–250 ml) and angioplasty (**B**) 100 ml (20–350 ml; p = 0.01).

Between September 2010 and April 2012 706 study participants (317 female) were included. 276 patients (39.1%) were diabetic, 605 patients (85.7%) had hypertension and 559 patients (79.3%) had an underlying heart disease defined as any history of ischemic heart disease, congestive heart failure, cardiac valvular defects or chronic cardiac arrhythmias. The median plasma creatinine at baseline was 1.20 (0.58–3.10) mg/dl corresponding to a median eGFR of 52.0 (15–100) ml·min^−1^·1.73 m^−2^. 437 (61.9%)  patients underwent a PCI, 269 (38,1%) had a percutaneous transluminal angioplasty or angiography of peripheral arteries, renal arteries or carotids, respectively (Table 1). From the 706 patients included, 296 underwent sole angiographic imaging receiving 95 ml (15–250 ml) of contrast media. The other 376 patients underwent angioplastic procedures receiving 100 ml (20–350 ml) of contrast media (p = 0.01, Fig. 1). 490 (69.4%) patients had an  eGFR lower or equal to 60 ml·min^−1^·1.73 m^−2^.

**Table 1:** Baseline characteristics and demographic data of all patients (*n* = 706).

Variable	Mean/median (±SD/range)
Age, y	74.0 (37–91)
Female, n (%)	317 (44.9)
Body mass index, kg/m²	27 ± 4.3
Hypertension, n (%)	605 (85.7)
Diabetes, n (%)	276 (39.1)
Heart disease, n (%)	559 (79.3)
Baseline MAP, mmHg	98 (57–140)
Percutaneous coronary angiography/intervention, n (%)	437 (61.9)
Other vascular angiography/angioplasty, n (%)	269 (38.1)
Contrast volume, mL	100 (15–350)
NSAID, n (%)	28 (3.97)
RAAS-blocker, n (%)	544 (77.1)
Diuretics, n (%)	422 (59.8)
Serum creatinine, mg/dl	1.30 ± 0.42
eGFR (MDRD),ml·min^−1^·1.73 m^−2^	52.00 ± 15.00
Cystatin C (mg/L)	1.20 ± 0.44
Urinary protein (mg/g Creatinine)	100 (0–7300)
Hospital stay (days)	2 (1–76)

*MAP*: mean arterial pressure, *NSAID:* nonsteroidal anti-inflammatory drugs, *RAAS:* renin angiotensin aldosterone system, *eGFR:* estimated glomerular filtration rate *(MDRD*: Modification of Diet in Renal Disease).

### Contrast induced acute kidney injury and outcome

**Figure 2:**
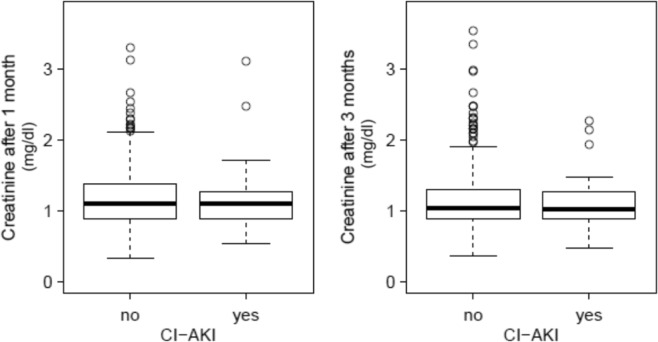
Serum creatinine after 1 month: CI-AKI: 1.15 ± 0.42 mg/dl; no CI-AKI: 1.18 ± 0.42 mg/dl (p = 59); Serum creatinine after 3 months: CI-AKI: 1.11 ± 0.34 mg/dl; no CI-AKI: 1.15 ± 0.42 mg/dl (p = 0.85).

**Figure 3:**
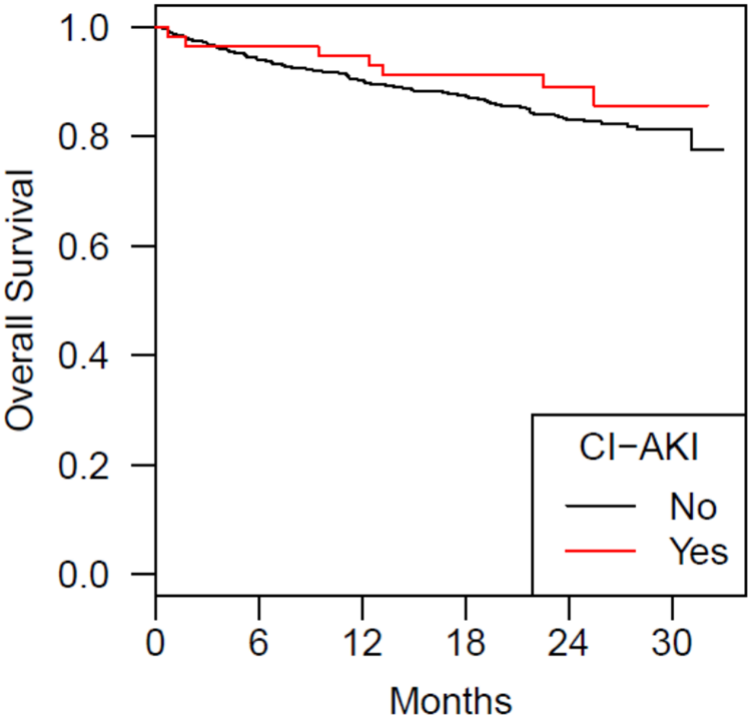
Overall survival after a median follow up of 25.5 months (n = 686): CI-AKI: 88.6%, without CI-AKI: 84.7% (p = 0.48).

From the whole cohort 72 (10.2%) patients developed a CI-AKI defined by a ≥25% increase of serum creatinine within 48 hours after contrast application. Using the KDIGO-criteria of AKI with an absolute increase of creatinine ≥0.3 mg/dl within 48 hours 66 patients (9.35%) were affected^15^. No patients had oliguria. Patients developing CI-AKI had a significantly better glomerular filtration rate at baseline than patients without CI-AKI (p = 0.007). The median eGFR was 57 (31–100) ml·min^−1^·1.73 m^−2^ and 52 (15–98) ml·min^−1^·1.73 m^−2^, respectively. Although patients undergoing angioplastic procedures received significantly larger volumes of contrast media than patients with sole angiographic imaging, both groups did not differ with respect to the CI-AKI incidence (p = 1.0, Table 2). Apart from baseline kidney function no other independent predictors of CI-AKI could be identified (Table 2). The pre-procedural saline did not result in a change of urine osmolality [median 470 (120–980) mOsm/kgH_2_O day −1 vs median 470 (130–920) mOsm/kgH_2_O day 0]. Plasma chloride levels dropped [median 103 (84–114) mmol/L day −1 vs median 102 (87–112) mmol/L day 1, p < 0.00001] post angiography. Patients developing CI-AKI were significantly longer hospitalized (median 3 days, range 1–35 days) than patients without CI-AKI (median 2 days, range 1–76 days; p = 0.014). After one month post angiography kidney function parameters of 407 patients and after 3 months data of 487 patients were available. In both instances serum creatinine was not different between patients with and without CI-AKI (Fig. 2). After a median follow up of 25.5 months 20 patients were lost to follow up. Of the remaining 686 patients all cause-mortality was not different between patients with (11.4%) and without CI-AKI (15.3%; p = 0.48, Fig. 3). The number of myocardial infarctions  was higher in the CI-AKI group, but no other differences with regard to cardiovascular events or causes of death between groups could be observed (Table 3).

**Table 2:** Predictors of CI-AKI.

Variable	Patients without CI-AKI (n = 634) Mean/median (±SD/range)	Patients with CI-AKI (n = 72) Mean/median (±SD/range)	*p-*value
Age,y	73 (37–91)	74 (47–89)	0.279
BMI, kg/m²	27 ± 4.4	27 ± 3.8	0.253
MAP _day−1_, mmHg	98 ± 13	97 ± 14	0.682
Contrast volume, ml	110 ± 51	120 ± 57	0.114
eGFR_day−1_, ml·min^−1^·1.73 m^−2^	51 ± 15	59 ± 15	<0.0001
Se-Creatinine _day−1_, mg/dl	1.3 ± 0.42	1.1 ± 0.29	<0.0001
Cystatin C _day−1_,mg/L,	1.2 ± 0.45	1.1 ± 0.36	0.111
Urinary protein _day−1_, mg/gCreatinine,	100 (0–7300)	120 (37–3100)	0.069
Urine osmolality _day−1,_ mOsm/kgH2O	470 (120–960)	470 (240–980)	0.906
**Sex (n,%)**			
Female	279 (44)	38 (52.8)	
Male	355 (56)	34 (47.2)	0.16
**Diabetes (n,%)**			
No	381 (60.1)	49 (68.1)	
Yes	253 (39.9)	23 (31.9)	0.19
**Heart disease (n,%)**			
No	381 (19.3)	8 (13.8)	
Yes	451 (80.7)	50 (86.2)	0.31
**Hypertension (n,%)**			
No	92 (14.5)	9 (12.5)	0.64
Yes	542 (85.5)	63 (87.5)	
**Diuretics (n,%)**			
No	257 (40.5)	27 (37.5)	
Yes	377 (59.5)	45 (62.5)	0.62
**RAAS-Blocker (n,%)**			
No	147 (23.2)	15 (20.8)	
Yes	487 (76.8)	57 (79.2)	0.65
**Statins (n,%)**			
No	112 (37.7)	10 (27.0)	
Yes	185 (62.3)	27 (73.0)	0.2
**Localisation (n,%)**			
Coronary	386 (60.9)	51 (70.8)	0.22
Peripheral arteries	230 (36.3)	19 (26.4)	
other	18 (2.84)	2 (2.78))	
Angiography (n,%)	296 (89.7)	34 (10.3)	
Angioplasty (n,%)	338 (89.9)	38 (10.1)	1.0

**Table 3:** Mortality and cardiovascular events after 2 year follow up; 20 patients were lost to follow up.

Cardiovascular event	Patients without CI-AKI (n = 616) n (%)	Patients with CI-AKI (n = 70) n (%)	*p-*value
Myocardial infarction	12 (3.3)	5 (12.8)	0.02
Heart failure	51 (14.2)	4 (10.3)	0.64
Repeat revascularization (coronary, peripheral)	97 (26.9)	11 (28.2)	1.0
Amputation	16 (4.4)	1 (2.6)	1.0
Ischemic stroke	13 (3.6)	1 (2.6)	1.0
Cerebral hemorrhage	3 (0.8)	0 (0)	1.0
Cardiac death	41 (6.7)	4 (5.7)	1.0
Cerebrovascular death	11 (1.8)	0	0.61
Other causes of death	44 (6.5)	3 (4.3)	0.46

### Decrease of creatinine (“Anti-CI-AKI”)

In 94 (13.3%) patients serum creatinine decreased ≥25% either 24 or 48 hours after contrast application. These patients had a significantly worse glomerular filtration rate at baseline than patients without Anti-CI-AKI (p = 0.007). A decrease of creatinine ≥0.3 mg/dl was observed in 89 (12.6%), a decrease ≥0.5 mg/dl in 26 (3.68%) patients within 48 hours after angiography. Significantly more patients experienced a 25% decrease of creatinine on both days after angiography (6.94%) than a 25% increase (2.41%, p = 0.0001).

## Discussion

The main finding of this prospective observational study in a high risk cohort was that the occurrence of CI-AKI had no adverse effects on mid-term kidney function, cardiovascular events or long term mortality. Our findings are therefore opposed to studies reporting that in 18–50% of patients CI-AKI is associated with a persistent increase of serum creatinine on the one hand^16–19^ and with long-term cardiovascular events and mortality on the other^3,18,20^. In our cohort 10.2% of patients developed a CI-AKI after receiving intra-arterial contrast media, a number in good agreement with recent reports^21–25^. In addition, we could not identify so far acknowledged risk factors such as old age, diabetes, contrast volume, proteinuria and renal impairment as being predictive for the development of CI-AKI. This somewhat counterintuitive finding is probably due to a risk minimization by exclusion of acutely ill patients, strict pre-procedural hydration and the sole use of low volume monomeric, low osmolar contrast material^26^. Moreover, there are several studies which came to quite similar conclusions questioning a compelling link between risk factors such as diabetes and CKD and the development of CI-AKI^8,27,28^. Patients with CI-AKI exhibited a significantly better kidney function at baseline than subjects without CI-AKI whereas patients with a decrease of creatinine after angiography had a worse glomerular filtration rate at baseline. These unexpected observations are possibly due to the regression to the mean phenomenon. Serum creatinine has substantial within-subject variability over time. Therefore, if it is extreme on its first measurement, it will tend to be closer to the average on its second measurement^29,30^. As the number of patients experiencing a decrease of serum creatinine on both days after contrast exposure outweighed the ones with an increase, our data give further support to the view that background fluctuation of kidney function interferes with a suspected diagnosis of CI-AKI^31–33^. Hospitalized patients with the need of an intra-arterial procedure such as coronary angiography have a high prevalence of established atherosclerotic disease. This group of patients often exhibit a fluctuating kidney function and are susceptible for developing an acute kidney injury of any cause^34^. Beyond that, several trials on patients undergoing i.v. contrast-enhanced procedures revealed that the frequency and magnitude of serum creatinine changes in subjects who have not undergone contrast-enhanced imaging does not differ from changes of patients who did receive contrast material. It is therefore simply not possible to unequivocally ascribe postangiographic changes of serum creatinine to the sole effect of radiocontrast agents^32,35,36^. Although CI-AKI has been associated with a long-term decline in kidney function^2^, in our study patients with and without CI-AKI did not differ with respect to their kidney function one and three months after angiography. This observation is in line with a very recent large prospective randomized trial revealing a very low risk for persistent kidney impairment in patients with CI-AKI^25^. After a two year follow-up we observed a higher number of myocardial infarctions in the CI-AKI group without any other differences in cardiovascular events. All-cause mortality and cardiovascular mortality was also not different between both groups. In a previous prospective observational study, the occurrence of CI-AKI after coronary angiography was associated with higher rates of 8-year cardiovascular adverse events^3^. However, in that study a high proportion of acutely ill patients in an emergency setting were included thus critically influencing overall outcomes. Recent literature affirms that the risk for CI-AKI is significantly determined by the presence and types of comorbidities and the acuity of illness^33^. Our cohort, though representing a high cardiovascular risk population, consisted of clinically stable patients undergoing planned angiographic procedures. In such a setting the risk for developing CI-AKI and its adverse sequelae, even in the presence of CKD, seems to be quite moderate^8,25,33^. Therefore, necessary diagnostic studies and interventions should not be withheld from patients with CKD due to some unfounded fear of CI-AKI.

In a recent clinical trial comparing balanced crystalloids with saline among patients treated in an emergency department, intravenous saline led to a significant increase of serum chloride concentrations paralleled by a decrease of bicarbonate 24 hours after application^37^. Although similar volumes of saline were used in our study, we could not observe any significant changes of chloride concentrations after saline infusion and therefore no safety concerns are warranted in that respect.

Our study has certain limitations. It has all the inevitable methodological shortcomings of a single center observational study. Furthermore, like most of the published trials on CI-AKI, we did not have a control group of patients not receiving contrast material. The two groups that were compared (CI-AKI vs. Non-CI-AKI) considerably differed in their size and statistical imbalances therefore cannot be ruled out. However, we think that if contrast application indeed had any relevant adverse impact it would have become apparent also in such a relatively small group. In addition, our findings are in line with many comparable studies of larger size hence confirming the plausibility of our results. Finally, due to our definition of CI-AKI using a rise in serum creatinine ≥25% within 48 hours after contrast application, it is possible that we missed CI- AKI cases occurring at later time points. This applies, however, to most of the CI-AKI studies.

In conclusion, in this prospective observational study we could demonstrate that in cardiovascular high risk patients including a high proportion of CKD-patients the risk of CI-AKI after intra-arterial angiography is quite moderate. The occurrence of CI-AKI had no significant impact on mortality and the numbers of cardiovascular events during a two year follow up period. Necessary contrast enhanced studies and interventions should not be withheld out of an exaggerated fear of CI-AKI.
